# Drone Mission Definition and Implementation for Automated Infrastructure Inspection Using Airborne Sensors

**DOI:** 10.3390/s18041170

**Published:** 2018-04-11

**Authors:** Juan A. Besada, Luca Bergesio, Iván Campaña, Diego Vaquero-Melchor, Jaime López-Araquistain, Ana M. Bernardos, José R. Casar

**Affiliations:** 1 Information Processing and Telecommunication Center, Universidad Politécnica de Madrid, 28040 Madrid, Spain; besada@grpss.ssr.upm.es (J.A.B.); luca.bergesio@grpss.ssr.upm.es (L.B.); icampana@grpss.ssr.upm.es (I.C.); diego.vaquero@grpss.ssr.upm.es (D.V.-M.); jaime.lopez@grpss.ssr.upm.es (J.L.-A.); jramon@grpss.ssr.upm.es (J.R.C.); 2 C-315.1, ETSI Telecomunicación, Avenida Complutense 30, 28040 Madrid, Spain

**Keywords:** unmanned aerial vehicles, mission planning, measurement planning, human-computer interfaces, multi-rotor trajectory prediction, infrastructure inspection

## Abstract

This paper describes a Mission Definition System and the automated flight process it enables to implement measurement plans for discrete infrastructure inspections using aerial platforms, and specifically multi-rotor drones. The mission definition aims at improving planning efficiency with respect to state-of-the-art waypoint-based techniques, using high-level mission definition primitives and linking them with realistic flight models to simulate the inspection in advance. It also provides flight scripts and measurement plans which can be executed by commercial drones. Its user interfaces facilitate mission definition, pre-flight 3D synthetic mission visualisation and flight evaluation. Results are delivered for a set of representative infrastructure inspection flights, showing the accuracy of the flight prediction tools in actual operations using automated flight control.

## 1. Introduction

In the last decade, we have assisted to a continuous market-growth and diffusion of unmanned aerial vehicles (UAV, drones), due to the important cost reduction of the involved technologies. Infrastructure inspection, sensing, maintenance, security and precision agriculture are just some of these aerial platforms endless applications. In this scenario, the complexity of the missions (flight and measurement/data gathering specifications) drastically increases, requiring new techniques and tools to easily define and perform a flight. In this paper, we focus on the infrastructure inspection problem with multi-rotors, although the involved techniques and tools can be adapted for other applications.

The workflow to define an infrastructure inspection mission involves a client/user, who may benefit from an aerial view, and a pilot, who actually flies the drone following the guidelines and requirements imposed by the client. The definition of the mission is often done through an on-the-field meeting, where the client explains the goals of the flight(s) to the pilot, usually with the aid of a map, and they agree on a plan. From the point of view of the pilot, it is quite often hard to follow a not well-defined flight plan, inspecting an area or covering a field, without an accurate estimation of the time of flight, and without real-time feedback on the quality of the mission implementation.

In this paper, we describe a Mission Definition System (MDS) designed to help the client to easily and efficiently define a drone mission, and supports implementing it with autonomous flight, under pilot direct line-of-sight, so drone control recovery is possible in any unsafe situation. Through a web interface, the proposed system enables to define a mission by decomposing the infrastructure of interest into basic geometries, selecting the measurement (sensing) procedures to be carried out in each part of the inspection. Once the user has defined a mission, it is forwarded to a Mission Calculation Engine, which is in charge of executing the appropriate calculations to obtain a compatible flight plan and predict a compatible trajectory associated to that mission. A Flight Plan is a time-ordered list of orders that a drone has to complete to fulfil the designed mission (i.e., take off, go to a waypoint, then hover to take a picture, then reach a second waypoint, then hover again to take a picture, ..., finally land), while a trajectory is defined through the time-sampled dynamic state variation of the drone along time. This trajectory is predicted taking into account the orders in the Flight Plan, and a model of the dynamic behaviour of the drone. Once both Flight Plan and trajectory are calculated, they may be represented in the system interface so the user can analyse them, check the duration of the flight, and even reproduce a simulation of the drone flight in a 3D simulated environment to check its safety and efficiency.

The tool enables also the communication of the described flight plan to the pilots, through a mobile application, and after mission acceptance by the pilot, it also facilitates to automate the flight control: once the Flight Plan is calculated and accepted, it is translated to a file type that the drone flight control system (i.e., autopilot) can understand. This file is sent to the drone or the control app. Additionally, detailed instructions on the measurement processes to be performed by the drone’s sensors are forwarded to the drone, so that the measurement processes are performed in sync with the flight operations. Those instructions, and the corresponding Flight Plan, form the complete Measurement Plan of the mission. To implement the flight, the drone carries out the mission that has been defined and implemented, following a trajectory ideally similar to the predicted trajectory. Pictures, thermal images, videos, or other measures are taken according to the Measurement Plan. As it is evident, the aforementioned system, if correctly implemented, may enable to take advantage of the drone as a sensing tool in an application-oriented way much more effectively than through manual operation of the pilot and camera/sensor operators in the field.

When comparing the system against existing tools, the reader will note that our proposal enables the definition of complex missions in a much more visual way, using meaningful 3D views of the operation, and linking higher level user requirements on the inspections to extremely detailed flight instructions, using physically sound multi-rotor flight models.

## 2. Background

### 2.1. UAV Fields of Application

According to the European Drones Outlook Study [1], the market of the drones will explode in the next thirty years in the fields of agriculture, energy, public safety/security, e-commerce/delivery and mobility and transport. It is easy to find examples of exploitation of drones for different purposes in the literature. For instance, in [2] authors propose to use a drone to acquire multispectral images and the ground difference for precision agriculture. In the field of public safety and security, apart from the countless military applications, new sensors are being adapted for drones, such as the ones used in [3]: X-ray camera, IR camera and metal detectors. Regarding e-commerce and delivery, applications are still at the beginning. The heavy impact of the weight on the battery duration and consequently on the distance is still a problem to solve. Despite this, the delivery of small objects is already a reality. In [4] it is described a service of transportation of small medicines and blood in Africa using a fixed wing UAV. For the e-commerce there are several proposals of the big technology companies, such as the Prime Air service of Amazon [5]. The field of mobility and transport evolves more slowly due to safety constraints and technology limitations, but it is also moving towards possible applications such as air taxis, etc. [6].

Therefore, UAV are already being explored as useful tools in multiple civil scenarios [7]. In many cases UAV-based systems are required to deliver functionalities such as surveillance and reconnaissance, monitoring, mapping and photogrammetry, automatic fault detection or inventory tasks. This article focuses on supporting inspection missions, thus next there is a review on different example UAV-based inspection experiences for e.g., photovoltaic plants, environment monitoring, roads, cell towers, railway lines, mines and buildings. Evidently, there are many other inspection scenarios in which drones are starting to be applied [8], such as power lines [9,10], levees and embankments [11], confined spaces, ecology [12,13], wind turbines, cranes [14], real estate [15], etc. As the reader will notice, traditional inspection procedures in most of these cases are costly, time-consuming, repetitive, labor-intensive and require technical expertise. The use of UAV alleviates and improves maintenance and risk prevention processes.

Results in [16] show that the procedure of utilising UAV in the detection of different failures of photovoltaic modules is much faster and effective than when using traditional methods. Specifically, authors aim at measuring the deformation measurement of a large-scale solar power plant by using images acquired by a non-metric digital camera on board a micro-UAS. In [17] a technique is documented to detect hot spots in photovoltaic panels (which are among the defects that may cause the most destructive effects), by analysing the sequence of thermal images. In [18], different UAV are employed to inspect a photovoltaic array field, using diverse thermal imaging cameras and a visual camera. The state-of-the-art in the Computer Vision field applied to photovoltaic plant inspection and to thermal anomalies detection over the panels is summarised in [19]. In addition, this paper present different data sets.

Monitoring of environmental gases for risk assessment both indoors (gas leaks, fires, mining applications…) and outdoors (agriculture biomass burning emissions, chemical and biological agent detection studies…) may require long periods of observation and large number of sensors. UAV may substantially complement existing ground sensor networks. For this purpose, an UAV has to be equipped with sensors capable to determine volatile chemical concentrations and detect gas leakages. For example, authors in [20] describe the design of a Gas Sensing System ready to be mounted on any UAV. In [21], a UAV which carries an on-board camera and a carbon dioxide gas sensor is capable of performing autonomous gas sensing while simultaneously visually detecting predefined targets placed at locations inside a room. The system is ready to transmit the collected data real time to a Ground Control Station for visualisation and analysis through a web interface.

Soil pollution monitoring is another application of UAV technology. For example, a multisensor approach for copper detection is explained in [22], in which authors present a system able to predict copper accumulation points at plot scales, using a combination of aerial photos, taken by drones, micro-rill network modelling and wetland prediction indices usually used at catchment scales. UAV may also be used for inspection of contaminated areas, such as the fission reactors for leakage detection, storage areas of nuclear sources, or even in hazardous scenarios of nuclear disasters [23,24]. In [25] the focus is on delivering a system that facilitates surveying forests, mapping canopy gaps, measuring forest canopy height, tracking forest wildfires, and supporting intensive forest management. In marine ecology, UAVs may be used e.g., to produce very fine scale maps of fish nursery areas. Authors of [26] detail the procedure of aerial photos acquisition (drone and camera settings) and post processing workflow (3D model generation with Structure From Motion algorithm and photo-stitching).

Road inspection UAV-supported procedures may help to detect early signs of erosion and pavement distress. In literature, there are some studies focused on landslide detection and monitoring such as [27,28], and some applied to road ditches [11]. Authors in [29] present a methodology to obtain, automatically, information about the conditions of the highway asphalt pavement, from data collected through remote sensing using an UAV (Unmanned Aerial Vehicle) and specific image processing and pattern recognition techniques.

Railway infrastructure inspection is addressed in [30], where a camera-based sensing and control methods are applied to two use cases: the first one when the UAV performs infrastructure inspection in close, but difficult-to-access areas (such as long bridges or tracks separated from the road by, e.g., a river), and the second oriented to railway track following for the sake of recording the infrastructure (such as tracks, sleepers, points or cabling). Target detection is carried out through different image descriptors (SURF, SIFT, FAST, Shi-Tomashi) and the performance of edge detectors for line detection is also analysed. Power line detection has also been addressed through UAVs. For example, [31] presents a vision-based power line detection algorithm, which is tested with multiple backgrounds and weather conditions. In the energy sector, the use of drones for the maintenance of power lines and transmission towers is already widespread. New diagnosis techniques based on drone use are emerging to improve the detection of problems, such as the one proposed in [32]. Electric towers detection, localisation and tracking is the core of [33], in which a combination of classic computer vision and machine learning techniques is proposed. The patent [34] describes a cell tower inspection procedure in which an operator using a UAV and a processing device may create a model of the cell site to then compare it against models created in subsequent inspections to determine significant differences between both. 5G advances, secure IoT and swarms of UAVs are combined into an architecture in [35] to guarantee Service in Critical Infrastructures (distributed generation plants, energy transmission and distribution networks, such as electricity cables and electrical isolators, and natural gas/Liquefied Natural Gas, tanks, pumps and pipelines…).

UAV are also applied to construction management. As a particular application, safety inspection on construction sites is addressed in [36], in which a drone is used to provide visual assets to verify the safety checklists in two different projects. Building inspection is also a common application; e.g., the patent [37] defines methods to perform an inspection of a rooftop by using a UAV, to extract information from a damaged area. Authors in [23] propose an architecture to inspect a reactor (blankets and other elements inside) before Remote Handling Systems operations (which imply the reactor shutdown) by using drones. They specify the type of sensing units and of suitable multicopters.

The project iDeepMon [38] aims at enhancing shaft surveying technologies towards a fully UAV-based automated process, integrated into the overall control process of an autonomous mine. In this line, a real mine-adapted UAV successful experience is described in [39]. The environment poses very defined constraints (to fly on absence of GPS aid and at low speed, to withstand small shocks with the rocks, variable lighting, etc.). Authors describe the hybrid equipment that was designed, using a helium gas filled balloon, with remote controlled quadcopter propellers, powerful LED lighting, rechargeable batteries, remote controlled cameras, image stabiliser, and radio frequency transmitters for control and image visualisation.

Many drone applications require photogrammetric data capture of complex 3D objects (building, bridges, monuments, antennas, etc.). In [40], it is presented an operational pipeline tested for photogrammetry in an archeological site. Algorithms such as Structure-from-Motion and multi-view stereo image matching facilitate the generation of dense meshed point clouds. Authors of [41] detail an automatic flight mission planning tool, which generates flight lines while aiming at camera configurations, which maintain a roughly constant object distance, provide sufficient image overlap and avoid unnecessary stations, based on a coarse Digital Surface Model and an approximate building outline.

The mission type obviously determines the measurement procedure to be completed (it terms of flight type, sensing payload and measurements to take) [42]. All these applications need tools to accelerate and partially automate the creation of missions, the calculation of the optimal trajectories and the automatic execution of parts of the mission with the least human intervention, in order to obtain cost effective solutions. Although it is possible to automate the procedure for very specific cases, it is difficult to build a generalizable automated system, so the approach in this paper is to provide the human controller with primitives that enable the effective and rapid generation of missions.

### 2.2. Review of Tools for UAV Mission Definition

Nowadays there are many dedicated tools for the definition of missions for UAVs. For instance, drone manufacturers companies have developed their own tools. Parrot and DJI tools [43,44], coming from drone manufacturing market leaders, follow the same modus operandi:

- They allow the user to flight manually the drone or to establish a set of points that conform a path to be followed by the UAV.
- They rely in standard map technologies such as Google Maps and offer a 2D point of view.
- In both cases, the application permits to create a flight plan and to automatically upload it to a drone for an automatic flight. In the case of Parrot, they use the MAVLink standard, which is a protocol currently used by many drones [45,46], used not only to automate flight but also to automate measurement process.

The tools are designed to enable a fast and easy way for the users to interact with them, to the detriment of more complex scenarios and systematic inspection definition. Additionally, there are several problems with such tools:

- They are intended to be used with the drones of the manufacturer, they are not open to be used with any arbitrary drone.
- The interface is oriented to simple flight plans defined through a time-ordered waypoints sequence, and to manual definition of other data such as altitudes or heading.
- The DJI most advanced automatic flight creation and execution is only available for the professional version of the application and for the top-tier aircraft.

Other companies provide more advanced platforms (with web interface and mobile applications) that link clients and pilots under the sharing economy paradigm, specially devoted to specific applications and with associated tools for mission specification. Three examples of such platforms can be found in [47,48,49]. Specifically, [47] is focused on the creation of maps and 3D reconstruction. Ref. [48] is open to other kind of missions, but it is restricted to the US airspace. Finally, Ref. [49] only permits to define missions that involve using the camera, it does not include other sensors in the mission definition.

A lot of research has been performed in the field of trajectory predictors, specially focused on the prediction of commercial aircraft trajectories and the definition of means to describe missions, flight plans and trajectories, such as [50,51,52,53]. Those trajectory predictors are used for mission optimisation or as a support tool for air traffic controllers to enable safe and efficient operations. The resulting trajectories do not perfectly model pilot/aircraft behaviour, but they have detail enough for the intended application. The underlying idea is calculating flight paths and trajectories minimising the costs of a flight, following predefined routes, respecting no-fly zones, taking advantage of prevailing winds, and selecting ideal flight levels, and it has been addressed using dynamic programming [54], genetic algorithms [55], heuristic search algorithms [56,57], or a combination of both genetic algorithms and heuristic search [58].

Nevertheless, the dynamics problem (fixed wing vs multi-rotor), and the accuracy constraints of the application are quite different from those in the Air Traffic Management area. There are also examples of research in trajectory prediction for drones, as [59,60] and many others, depending on the type of UAV to control, and on the application. The academic ambit has focused recently on the goal of generalising the definition of the missions from a high level point of view, as explained for example in [61]. The authors developed a Domain Specific Language that enables setting mission specifications, similar to the aforementioned efforts in [51,53]. They achieved the capability to define more complex, richer and detailed behaviours, with the cost of a more difficult interaction with the user. This example is to a certain extent similar to our approach. It makes use of the open source platform for mission planning of autonomous quadrotor called FLYAQ, also described in [62]. It allows different types of movement and sensor control, translating from a high level specification of the mission into a Quadrotor Behaviour language (QBL), similar to the ones described for ATM applications. FLYAQ per se does not allow defining automatic inspections for different geometric forms nor 3D trajectory visualisation or planning, although the extension in [61] includes the possibility to define point, line (with intermediate waypoints), polygon and volume (polyhedral) primitives. This reference focuses in this part of the translation, while our proposal covers the whole planning system (going from mission to dynamically sound definition of detailed trajectory), while also describing associated 3D user interfaces. Our approach is also more complete in terms of mission primitives.

Other support tools to make possible for non-expert users (e.g., firefighters, rescue workers, etc.) to specify missions are also being delivered. It is the case of [62], in which the detailed flight plan that each multicopter must perform to accomplish the specified mission is automatically generated by preventing collisions between multicopters and obstacles, ensuring the preservation of no-fly zones. The teleoperator viewpoint is also considered in [63], through a specific interface that provides real-time environment-adaptive viewpoints, automatically configured to improve safety and smooth user operation. This facilitates to handle situations in which nearby objects can be collision hazards and frequent occlusion can hinder accurate manipulation. The system, which uses simultaneous localisation and mapping (SLAM)-based reconstruction, combines robot position and orientation, and 3D pointcloud information to modify the user viewpoint to maximise visibility.

In [64], the process for defining the mission of a specific system (the Neptus framework) is described. Its mission planning is focused on heterogeneous teams of vehicles such as autonomous and remotely operated underwater, surface, land, and air vehicles. It has a predefined set of manoeuvres and does not allow to generate automatic routes for static target inspection. As in the previous reference, in [65], a specific mission definition system that mixes manned and unmanned aircraft has been defined, with similar limitations on the use with commercial drones. Another mission path planning for UAVs, focused in the surveillance of moving targets, is described in [66]. The mission is calculated by minimising a cost function adapted to follow mobile targets, quite different to the static infrastructure inspection missions we are dealing with. Other types of mission planner are presented in [67], where the mission is recalculated in real time but again it is oriented to obtain a path to a non-static target, and in [68], specifically for pick up from the warehouse to the production line until the final product is delivered to the client. In [60] it is also described the flight plan definition problem as a concatenation of legs joining waypoints, which is not cost effective for typical systematic infrastructure inspection flights, and [69] describes a complete language hierarchy and associated calculation engines, mainly devoted to the lower levels of the trajectory prediction problem for commercial aircraft and also for multi-rotors.

Finally, an analysis of the requirements that must be met by a drone mission definition system can be found in [70], while an approach to define ways to increase the probability of success of the mission can be seen in [71]. Dynamic constraints should be taken into account, each drone has a minimum and a maximum observation altitude, and the drone’s energy consumption is related to this altitude. In [72], the problem of finding drone locations that minimise the cost while ensuring the surveillance of all the targets is addressed and solved by defining an integer linear model and a mixed integer non-linear optimisation model.

## 3. Materials and Methods

Next we will describe the overall architecture and implementation of the drone Mission Definition System (MDS), its main subsystems and interactions with the user in charge of defining the mission (to be called the MDS user), the drone pilot, and the drone autopilot.

This section has the following main subsections: Section 3.1 details the system high level requirements and the high level interactions with its users, the pilots and the autopilots; Section 3.2 describes the whole MDS architecture; Section 3.3 details the underlying input data model and the web/mobile interfaces enabling the user and pilot to develop a common view of the operation; Section 3.4 describes the algorithmic core of the system, including calculations enabling trajectory simulations, and the translation of the derived flight and measurement plans to a suitable instruction language for the autopilot (such as MAVLINK); and finally Section 3.5 describes the use of the aforementioned flight and measurement plans by the drone systems.

### 3.1. High Level Requirements and System Interactions

We next describe the workflow in the MDS ecosystem, understood as a system composed by a cloud platform (the MDS system) performing all the relevant calculations for mission definition by the MDS user and communication with the pilot and the autopilot, and its relevant interfaces, to be described next. Three types of actors appear in these interactions:

- The MDS users, assumed to be the clients of the MDS platform, in charge of designing an inspection operation, pre-visualising and, after qualifying it as satisfactory, forwarding it to the pilot assigned by the platform. They use a MDS web-based interface with the cloud platform.
- The drone autopilots or flight control systems, receiving the calculated flight plan to be automated in a relevant format (i.e., MAVLINK). In order to facilitate flight plan provision, the MDS cloud platform contains a connector to autopilot systems, using either automated or manual procedures to distribute the flight plans in the relevant format.
- The human pilots, receivers of the designed operation, and if accepted, with the responsibility to implement it (with the automated flight support by the autopilot). Their interface with the cloud platform is the pilot App, which is a mobile/web App used to distribute and visualise missions in the field.

The MDS system can work concurrently with a collection of operations/missions involving different MDS users, human pilots and autopilots, using secured communications and only giving access to each of the actors to the information related to their operations.

Next, the different use cases relevant to the MDS user will be described summarising the interaction with the MDS web-based HMI. The order of the use cases presented follows a typical mission life cycle. Inside each use case, the different stages (separated by a ;) are enumerated:

1. Define a Mission: Log into system; define the mission objectives; set the initial (take-off position) and the final (landing position) waypoints; request MDS system to generate flight plan and trajectory for the mission; mission gets saved in MDS User Mission list.
2. Modify a Mission: Very similar to the previous one, but selecting a mission to be edited. Objectives and initial and final waypoints may be edited.
3. Clone a Mission.
4. Visualise a Mission: log into system; select mission from MDS User Mission list; select visualisation view; and request MDS system to produce and export visualisation results (video, test, …). Visualisation view can be: Mission in 2D or 3D (or textual description), Flight Plan in 2D or 3D (or textual description), Flight Plan in suitable autopilot format (i.e., Mavlink), Trajectory in 2D or 3D, and Simulated Flight Video.
5. Approve a Mission: select a pilot from an available pilot list; select a previously defined mission from MDS User Mission list; send a notification to the pilot so he/she may decide to accept it; finally MDS system changes mission state to APPROVED.

Next the pilot use cases, implemented through its pilot App interface, are described for as typical mission life cycle:

1. Receive a Mission Notification: receive and preview it (some key information should be provided in notification).
2. Accept/Decline mission: log into the system; select a not DECLINED Mission from Pilot Mission list; and Accept/Decline it. As a result, the MDS system, changes mission state to ACCEPTED or DECLINED.
3. Visualise a Mission: It is completely equivalent to the Mission Visualisation MDS user use case, but implemented in the pilot App. Only not DECLINED Missions should be visible.
4. End mission execution: log into the system; select an ACCEPTED Mission from Pilot Mission list; and terminate it. As a result, the MDS system, changes mission state to END.

Additionally, the MDS cloud platform provides interfaces to distribute the calculated flight plan in autopilot/Flight Control Systems formats such as MAVlink. Automated retrieval from mobile apps for drone piloting is possible using a REST interface, and also, manual access to it may be obtained both through the MDS web HMI and through the Pilot App.

### 3.2. MDS Functional Architecture and Context

**Figure 1:**
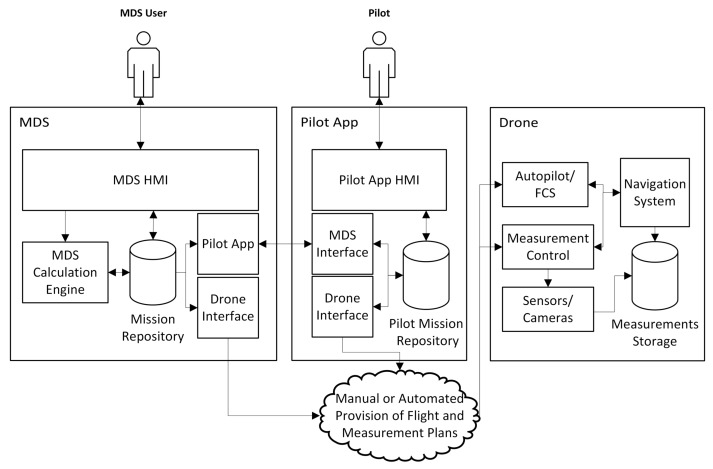
Mission Setup System.

The MDS ecosystem architecture is depicted in Figure 1. As can be seen there, it is composed of three main subsystems: the MDS system itself, the Pilot App, and the drone systems.

The MDS system is composed of several cooperating subsystems. The MDS system Web-based HMI (MDS HMI in Figure 1) implements MDS user use cases, making use of the underlying infrastructure. It may also give manual access to drone autopilot and measurement control automated operation specifications (i.e., MAVlink), for cases in which the drone system has no automated means to retrieve or receive this data. Meanwhile, the MDS Calculation Engine is the core of the MDS system, which generates the complete flight plan and measurement plan from the mission specification, predicting the flight according to expected aircraft dynamics, and saves it in a mission repository which can be accessed through the HMI and by the other components of the MDS system. The pilot App Interface System is in charge of providing the missions notifications and specifications to the pilot App, and managing the acceptance and termination of those missions by the pilot. The final MDS system component is the Drone Interface System, converting the flight and measurement plans in suitable formats for the autopilot/Flight Control System (FCS) and the measurement control system in the drone. In highly automated approaches, if the drone autopilot allows remote access, it provides the flight script directly to the drone systems at the beginning of the flight. The definition of the MDS system is the core of our contribution.

The Pilot App is also composed of several components. It has its own HMI (Pilot App HMI in the figure) implementing Pilot use cases. It may also give manual access to drone autopilot and measurement control automated operation specifications (i.e., MAVlink), if the drone is controlled from a mobile app receiving this kind of specification. Another component of the Pilot app is a MDS Interface System, in charge of receiving the missions notifications and specifications from the MDS system, and managing the acceptance of those missions by the pilot. Finally, there is a Drone Interface System converting the flight and measurement plans in suitable formats for the autopilot/FCS system and for the measurement control system in the drone. It is functionally equivalent to the one in the MDS system.

Finally, the drone itself should contain several systems to make effective use of the MDS results and automate both flight and measurement processes. First, the Autopilot/FCS system, making use of the flight instructions to control the aircraft dynamics. Then, a Navigation System, enabling the autopilot to perform its flight by controlling the error between the desired trajectory and the time-varying dynamic state of the drone. The Navigation System should also obtain time-stamped dynamic state samples, to be able to georeference the available measures. Additionally, the drone should have an on-board Measurement Control System, in charge of taking the measures at the appropriate locations, or after some instructions have been performed, in accordance with the measurement plan. Finally, the drone must also have a set of Sensors (optical still cameras, video cameras, thermal cameras, etc.), whose measurement process should be controlable by the measurement control system. The resulting measurements should be automatically stored with a time reference.

### 3.3. MDS Input Data Models and Associated Input HMI

This section details the MDS data model and its web interface. Starting with the data models, in order to allow the user to generate missions in an easy way, we have defined some basic inspections, enabling to build more complex mission specifications by concatenation. The definition of any basic inspection has three main parts: a definition of the geometrical shape to be inspected; a minimum and maximum drone distance to this shape (and sometimes also constraints on sensor orientation); and a measurement process specification.

We have defined a list of geometrical shapes (with any orientation in 3D): points; straight lines; polylines (defined as a list of consecutive straight lines); catenaries; polygons; circles; prisms (straight or oblique); pyramids (straight or oblique); cylinders (straight or oblique); truncated cylinders (defined as cylinders plus two truncation planes); cones (straight or oblique); and truncated cones (defined as cones plus two truncation planes). This list has been proved as a good initial set allowing to define typical infrastructure inspection missions. It could be extended in the future for new applications.

Each basic inspection may have one or several measurement processes. It details the sensor (or sensors) that must be used to perform measurements, and specifies a sampling process for the inspection. This basic inspection sampling process will result in the definition of a list of sampling positions and later on the calculation of associated waypoints. The next information must be provided for each sensor (with minor adaptations for each type of basic inspection):

- Sensor name: ID of the sensor to be used.
- Measurement duration: Time needed to complete all the measures at each sampling position.
- Measurement period: Time between two measures at each sampling position.
- Linear sampling distance: Linear distance between two sampling positions.
- Angular sampling distance: Angular distance between two sampling positions.

**Figure 2:**
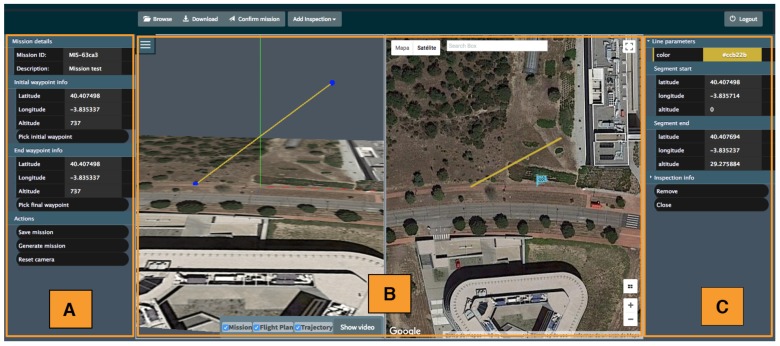
Main interface of the web tool.

The MDS HMI, that can be used in any up-to-date web browser, is depicted in Figure 2. Its layout is divided into three sections/panels, as follows:

A. Mission details: This panel contains the general information associated to the current mission: the mission ID, a brief text description of the mission and the initial and final waypoints. It also contains buttons to manage missions status (save it, generate flight plan and trajectories, etc.).
B. 3D View and Map View: This central section is divided into two subsections arranged horizontally. The left one corresponds to the generated 3D View, using WebGL technology to generate/manipulate 3D views of the infrastructures, missions, flight plans and trajectories. This 3D view changes according to the center and scale of the 2D map from Google Maps, located at the right of the panel.
C. Inspection details: This panel holds the details of a selected inspection within the mission specification. The information and parameters contained in this panel change in function of the inspection type.

Details about the tool usage in a realistic inspection example are provided in the Section 4.1.

### 3.4. Mission Calculation and Associated Data Models

**Figure 3:**
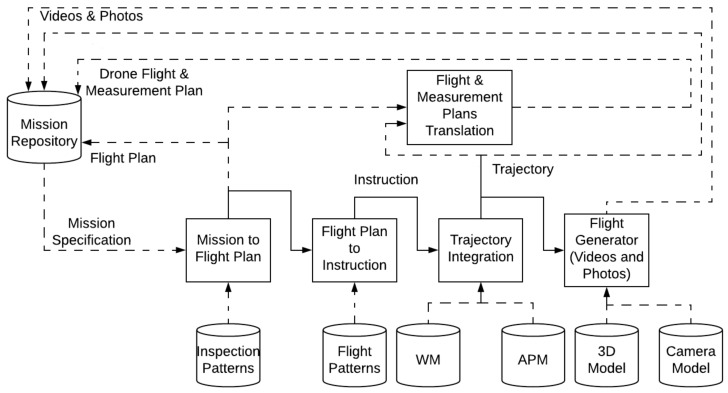
Architecture Mission System.

This section describes the internal calculations of the Mission Calculation Engine, whose internal architecture is depicted in Figure 3. It receives the mission specification as input and provides as output a Flight Plan, the associated Trajectory, a specification allowing the generation of virtual scenario videos and photos to be visualised in the MDS HMI or the Pilot App, and flight scripts (and Measurement Plans) in drone specific formats (i.e., MAVlink). The process may be decomposed sequentially in several phases.

First the Mission specification (list of basic inspections) is converted into a Flight Plan. Following predefined inspection patterns it is possible to generate a list of waypoints (3D coordinates and bearings to illuminate sampling points), enabling to complete the measurement programme. The waypoints list constitutes the flight plan. Section 3.4.1 is devoted to a detailed description of the generation of a Flight Plan from a Mission specification.

Then, the Flight Plan is translated into a sequence of instructions modelling the operation of the drone to fulfil the Flight Plan requirements. Those instructions should not be confused with those finally issued to the drone, as they are of a higher level, and they are used to simulate the constraints on the dynamic state of the drone imposed by its guidance and control systems. This translation makes use of predefined flight patterns summarising flight operational behaviour, and also takes into account the on-board sensors features, to define the time intervals where the drone has to remain still on specific coordinates to complete the measurement schedule. The instructions are defined in a QR-AIDL inspired manner [69], and they are not drone specific. This sequence of instructions is sent to the Trajectory Integration Engine in order to obtain the sampled trajectory. The computation of an accurate trajectory requires to consider the following additional information: (i) the drone physical features, such as its weight, motors thrust, aerodynamical performance, etc. which are part of the Aircraft Performance Model (APM); (ii) the weather model information, to introduce the potential effect of the wind (WM). From these data, the trajectory integration engine solves a system of differential equations modelling drone motion dynamics and obtains all the relevant parameters that model the trajectory. Section 3.4.2 details the calculations to generate a trajectory from the flight plan.

The trajectory is then used to derive a simulated video of the flight or a set of simulated pictures, making use of 3D models and camera models. Section 3.4.3 describes the final calculations necessary to provide these meaningful representations of the trajectories to the MDS user and to the human pilot.

Finally, the Flight Plan and Trajectory are translated into drone specific flight and measurement plans. The instructions in this case are of much lower level, and different translators for different formats are needed. This translation is detailed in Section 3.4.4.

#### 3.4.1. Flight Plan Calculation

To build the Flight Plan, the initial Waypoint (take-off position), final Waypoint (landing position) and the list of basic inspections described in Section 3.3 are processed. The complete Flight Plan is built by first creating partial flight plans associated to each basic inspection, which are then merged. Next we will detail the process to build a partial flight plan, for an example straight cylinder inspection. Similar processes are performed for the rest of the basic inspections. All coordinates in the following description are local horizontal, (X-East, Y-North) Stereographic Cartesian, although in the HMI all geographic data is provided through latitude, longitude and height with respect to the local horizontal. These input data are transformed into XY using a stereographic projection centred in take-off position.

In the case of a straight cylinder, five geometrical figures may be inspected (each of them with independent sampling processes specifications):

- Bottom Base Circumference and Top Base Circumference, sampled at constant angular distance.
- Bottom Base Circle and Top Base Circle, sampled using polar coordinates at constant angular and linear sampling distances.
- Cylinder Wall, sampled using constant angular and height-aligned linear sampling distances. This is the sampling process to be detailed next.

**Figure 4:**
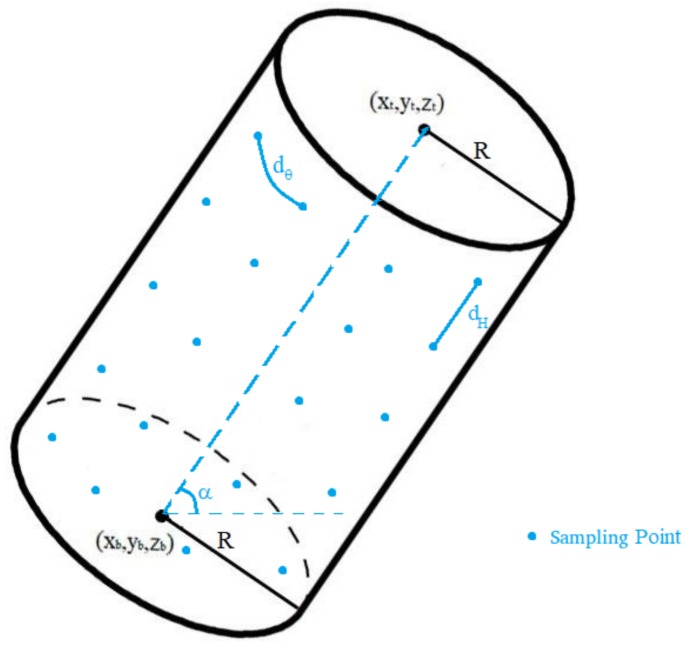
Cylinder parameters and sampling points.

The input data for the process to derive the sampling points for the cylinder wall would be: Cylinder Radius (*R*); Bottom Base 3D center (${\vec{x}}_{b}=\left(x_{b},y_{b},z_{b}\right)$); Top Base 3D center (${\vec{x}}_{t}=(x_{t},y_{t},z_{t}$); Maximum drone separation to the cylinder ($d_{max}$); Minimum drone separation to the cylinder ($d_{min}$); Linear Sampling distance ($d_{H}$), in meters, used for height dimension; and Angular sampling distance ($d_{\theta }$), in radians. In Figure 4 some of the cylinder parameters are depicted, for an example cylinder. Also, the resulting sampling points are depicted in blue. Those points are the result of the cylinder wall sampling process, to be described next.

First of all, a cylinder wall is inspected as a set of circles at different heights. The number of circles ($n_{circles}$) sampling the cylinder is:

(1) $$n_{circles}=\frac{\sqrt{{\left(x_{t}-x_{b}\right)}^{2}+{\left(y_{t}-y_{b}\right)}^{2}+{\left(z_{t}-z_{b}\right)}^{2}}}{d_{H}}$$

The sampling points are calculated, in a reference frame centred in the bottom circle cylinder, with height axis along the cylinder axis. To do so, we first calculate the polar coordinates of those points ($\left(R,\theta_{i},H_{j}\right)$, with i and j indexes, where i goes from 0 to $2\pi /d_{\theta }$, while j goes from 0 to $n_{circles}$), as ($\left(R,\theta_{i},H_{j}\right)=\left(R,id_{\theta },jd_{H}\right)$). Next we convert these sampling points to Cartesian coordinates in the previous reference frame ($(x_{i,j}$, $y_{i,j}$, $z_{i,j})=\left(R\cos\left(\theta_{i}\right),R\sin\left(\theta_{i}\right),H_{j}\right)$). Each of the sampling points need to be rotated and translated to local horizontal XYZ centred in take-off position. For this it is necessary to know the elevation (α) and the heading (β) of the cylinder inspected. These two angles are obtained from the vector difference of the circles centres ($\vec{d}=\left(d_{x},d_{y},d_{z}\right)=\left(x_{t}-x_{b},y_{t}-y_{b},z_{t}-z_{b}\right)$), as:

(2) $$\begin{array}{c} \alpha =\frac{\pi }{2}-\arctan\left(\frac{\sqrt{d_{x}^{2}+d_{y}^{2}}}{d_{z}}\right)\\ \beta =\arctan\left(\frac{d_{y}}{d_{x}}\right) \end{array}$$

Finally, the sampling points are rotated and translated to local horizontal XYZ centred in take-off position ($x_{s}\left(i,j\right)$, $y_{s}\left(i,j\right)$, $z_{s}\left(i,j\right)$):

(3) $$\left[\begin{array}{c} x_{s}\left(i,j\right)\\ y_{s}\left(i,j\right)\\ z_{s}\left(i,j\right) \end{array}\right]=\left[\begin{array}{ccc} 1 & 0 & 0\\ 0 & \cos\left(\alpha \right) & -\sin\left(\alpha \right)\\ 0 & \sin\left(\alpha \right) & \cos\left(\alpha \right) \end{array}\right]\left[\begin{array}{ccc} \cos\left(\beta \right) & -\sin\left(\beta \right) & 0\\ \sin\left(\beta \right) & \cos\left(\beta \right) & 0\\ 0 & 0 & 1 \end{array}\right]\left[\begin{array}{c} x_{i,j}\\ y_{i,j}\\ z_{i,j} \end{array}\right]+\left[\begin{array}{c} x_{b}\\ y_{b}\\ z_{b} \end{array}\right]$$

Once we calculated the sampling points, there are many potential strategies for the generation of waypoints. In our current implementation, we have three different simple approaches:

- Normal to surface illumination.
- Defined constant camera tilt and drone yaw angles.
- Defined constant camera tilt, normal to surface drone yaw angle.

For some of the basic inspections (i.e., point, line, catenary) only some of these strategies may be used, as others would lead to ambiguous definition of the waypoint position. An implicit assumption in our model is sensor gimbal does not rotate horizontally (pan angle is set to zero), so that the line of sight direction is controlled by drone yaw angle. This simplifies flight plan calculation and modelling, and results in simplified payload with reduced weight and operation control.

Next we will describe the results of the Normal to surface illumination strategy for the cylinder wall, as an example. From each sampling point, a waypoint is calculated in perpendicular direction to the cylinder wall, at a distance between $d_{min}$ and $d_{max}$. We first define a difference vector from the bottom center to the (*i*,*j*) inspection sampling point ($\vec{d_{i,j}}=\left(d_{x}\left(i,j\right),d_{y}\left(i,j\right),d_{z}\left(i,j\right)\right)=\left(x_{s}\left(i,j\right)-x_{b},y_{s}\left(i,j\right)-y_{b},z_{s}\left(i,j\right)-z_{b}\right)$). The normal vector (${\vec{n}}_{i,j}=\left(n_{x},n_{y},n_{z}\right)$) may be calculated, using cross vector product, as:

(4) $${\vec{n}}_{i,j}=\left(\vec{d}\times \vec{d_{i,j}}\right)\times \vec{d}$$

To obtain the waypoints positions we move along this normal vector a distance equal to the average between the minimum and maximum distance to the inspection ($d_{med}=\frac{d_{min}+d_{max}}{2}$). Therefore, waypoint position ($x_{W}\left(i,j\right),y_{W}\left(i,j\right),z_{W}\left(i,j\right)$) results:

(5) $$\begin{array}{c} x_{W}\left(i,j\right)=x_{s}\left(i,j\right)+d_{med}\frac{n_{x}}{\sqrt{{n_{x}^{2}+n_{y}^{2}+n_{z}}^{2}}}\\ y_{W}\left(i,j\right)=y_{s}\left(i,j\right)+d_{med}\frac{n_{Y}}{\sqrt{{n_{x}^{2}+n_{y}^{2}+n_{z}}^{2}}}\\ z_{W}\left(i,j\right)=z_{s}\left(i,j\right)+d_{med}\frac{n_{z}}{\sqrt{{n_{x}^{2}+n_{y}^{2}+n_{z}}^{2}}} \end{array}$$

If the MDS user instead prefers constant camera tilt (ϕ) and drone yaw (θ) angles, the associated waypoint is calculated as follows. First, the unitary vector ($\vec{u}$) from the sampling point to the waypoint is calculated ($\vec{u}=\left(u_{x},u_{y},u_{z}\right)=\left(-cos\left(\theta \right)cos\left(\phi \right),-sin\left(\theta \right)cos\left(\phi \right),-sin\left(\phi \right)\right)$). Then, a point (to be called ${\vec{x}}_{1m}\left(i,j\right))$, situated at one meter from the sampling point in the direction of the aforementioned unitary vector, is calculated as ${\vec{x}}_{1m}\left(i,j\right)={\vec{x}}_{s}\left(i,j\right)+\vec{u}$. This point is hopefully further than the sampling point from the cylinder axis. Next we calculate the distance of ${\vec{x}}_{1m}$ to the cylinder axis, using the cross vector product, as:

(6) $$R_{1m}\left(i,j\right)=\frac{∥\left({\vec{x}}_{1m}\left(i,j\right)-{\vec{x}}_{b}\right)\times \vec{d}∥}{∥\vec{d}∥}$$

The distance increment from ${\vec{x}}_{1m}\left(i,j\right)$ to ${\vec{x}}_{s}\left(i,j\right)$ point to the cylinder axis would be $R_{1m}\left(i,j\right)-R$. If this value is negative, the waypoint cannot be calculated. If it is positive, to increment the distance to the axis to $d_{med}$ (calculated again here as $d_{med}=\frac{d_{min}+d_{max}}{2}$), the waypoint needs to be located in:

(7) $${\vec{x}}_{W}\left(i,j\right)={\vec{x}}_{s}\left(i,j\right)+\frac{d_{med}}{R_{1m}\left(i,j\right)-R}\vec{u}$$

**Figure 5:**
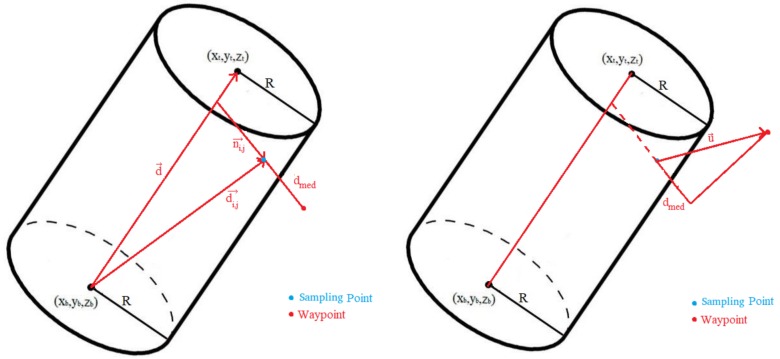
Input data to get the waypoints: (**left**) orthogonal, (**right**) constant tilt and yaw.

The resulting waypoints, with orthogonal illumination of the cylinder sampling can be seen in the left of Figure 5, while the waypoints obtained using constant camera tilt and drone yaw angles are depicted in the right of this figure.

If the illumination strategy is based on a defined constant camera tilt, normal to surface drone yaw angle, the yaw angle is first calculated making use of the calculation of a vector normal to cylinder wall surface (as described in Equation (4)), followed by the calculations described in previous steps for constant tilt and yaw angles illumination strategies, using the desired tilt and the previously calculated yaw.

Similar algorithms need to be executed for the different basic inspection shapes, sampling patterns, and illumination strategies.

Finally, it is clear that depending on the surface to be inspected and on the calculated waypoints the result of the previous processes may present additional problems. For instance, using normal illumination from below a horizontal wall results in the sensor being pointed upwards, and in the drone being just in the middle of the line of sight of the sensor (precluding correct measurement taking). In some other cases the waypoint is too close to an obstacle, inside it, or its line of sight occluded by it (imagine for instance a prism with constant tilt and yaw: only some of its faces will be visible in that direction). Therefore, after calculating all sampling inspection points and associated waypoints, the resulting data must pass through a visibility/safety test. This test takes into account all inspection elements and other obstacles defined in the mission. The parts of the inspection which are not attainable given the visibility/safety constraints are marked for evaluation, so the MDS user may change the flight definition. So there will be a collection of waypoints, with their associated sampling points (and therefore drone yaw and camera tilt angles), and a set of measurement processes which specify if the drone has to be stopped while taking a sensor measure and how long it will take to do so. To derive from this data a Flight Plan, the system just needs to decide the waypoint visit order. This may be done using two kinds of strategies: searching for an efficient ordering (i.e., shortest path length, using a Dijkstra or similar algorithm), or making use of predefined visit order strategies.

This second strategy is the one followed in our prototype, where the underlying goal is to facilitate manual control by minimising the number of control variables to be modified in flight legs (movement between waypoints). Therefore, for instance, to explore vertical cylinders, visiting patterns with vertical legs are in general preferred to horizontal legs, as the former just request altitude control (1 variable), while the later needs controlling at least 2 variables (X and Y horizontal position), if not 3 (also yaw, if normal yaw is used as illumination strategy). Different visiting strategies are defined for different basic inspections and different orientations of the inspection volume. Even in this case, in order to connect the basic inspection in a Flight Plan, length minimisation of the legs joining basic inspection visiting patterns should be performed.

#### 3.4.2. Trajectory Calculation

From the Flight Plan and measurement plan (time ordered waypoints, with associated drone yaw, camera tilt and measurement process), the MDS system next derives a simulated trajectory. It is calculated to: (i) give the MDS user feedback on mission implementation, to enable their iterative definition; (ii) assess in advance the capability of the drone to perform the complete flight or the need to split it into several flights, by assessing battery consumption; (iii) give the human pilot every detail on the expected trajectory, so enabling monitoring it and performing a handoff to manual control if something unexpected happens; (iv) and finally to support the creation of simulated videos of the flight to enhance pilot and MDS user awareness of the intended operation (as detailed in Section 3.4.3).

To do so the Flight Plan is decomposed in simpler elements capable of simulating the flight along legs joining consecutive waypoints. Keeping the original idea of the QR-AIDL [69], we denoted those simpler elements *instructions*. Instructions are defined through physical laws that allow to model the drone movement, completed with an end condition (for instance, arriving to a given position or reaching a defined speed). These laws are translated into mathematical expressions constraining the trajectory kinematic parameters. Specifically, the following parameters are modelled to constrain the trajectory in our system: horizontal groundspeed ($\left(v_{x},v_{y}\right)=\left(\frac{dx_{e}}{dt},\frac{dy_{e}}{dt}\right)$), vertical speed ($v_{z}=\frac{dz_{e}}{dt}$) and yaw (ψ), all expressed with respect to the earth-fixed frame. Constraining these parameters, all the remaining parameters are fixed with physical laws, as it will be later explained. Then, the instructions are a set of expressions for the parameters $\left(v_{x},v_{y},v_{z},\psi \right)$ allowing any kind of manoeuvre. With a sequence combining these instructions the manoeuvre is defined in an unambiguous way: from it we may calculate the drone 3D position ($x_{e},y_{e},z_{e}$) at any time, and also the (ϕ,θ,ψ) rotation angles (Euler angles) around the Cartesian axes.

For example, Equation (8) shows the mathematical expressions for a constant groundspeed, constant vertical speed and fixed yaw (ψ(t)) instruction. In the equation, the values $k_{i}$ are real constants constraining the speed and yaw along this part of the flight. A potential end condition for the instruction could be $x_{e}=0$, meaning those constraints will be in effect until the drone X position reaches 0.

(8) $$v_{x}\left(t\right)=k_{1};v_{y}\left(t\right)=k_{2};v_{z}\left(t\right)=k_{3};\psi \left(t\right)=k_{4}$$

Another example instruction, modelling the start of a vertical manoeuvre, could be defined through mathematical expressions for a zero groundspeed, constant vertical acceleration ($k_{a}$, from instant $t_{0}$) and fixed yaw ($k_{4}$), as:

(9) $$v_{x}\left(t\right)=0;v_{y}\left(t\right)=0;v_{z}\left(t\right)=k_{a}\left(t-t_{0}\right);\psi \left(t\right)=k_{4}$$

An end condition for this instruction might be $v_{z}\left(t\right)=v_{max}$, meaning the previous constraints will remain effective until the drone vertical speed reaches $v_{max}$.

A concatenation of adequate consecutive instructions may model the different guidance patterns used by the drone to implement a given leg. For instance, a straight horizontal leg with stopped start and end states, and with a given target yaw, could be implemented through a series of accelerate-keep speed-decelerate-turn instructions, where the acceleration instruction need also to define the heading of the movement (to be kept for the whole leg). Meanwhile, a straight vertical leg with no change in yaw would be implemented by a series of vertical accelerate-keep speed-vertical decelerate. These series of instructions implementing a leg need to fulfil three requirements. First and foremost, they have to respect the initial and ending conditions, meaning, that they have to start and end into the waypoint positions defined in the Flight Plan. Second, all the drone physic parameters have to be continuous along the trajectory. Finally, all dynamic constraints should be taken into account, and specifically the maximum rotation speed of the motors need to be respected. This later point may impose changes on some of the instruction parameters (for instance, too high speeds cannot be attained).

After obtaining the instructions, a high fidelity trajectory prediction engine is in charge of transforming them into a predicted set of positions. Using QR-AIDL underlying model, the motion of a quadrotor is that of a point mass where its orientation (Euler angles) and the sum of all propellers forces are the control inputs. This leads to a simplified model with four mechanical degrees of freedom (closed for each time by the four constraints in the instruction). The model can be written as:

(10) $$\frac{d^{2}x_{e}}{dt^{2}}=\frac{\left(\cos\phi \sin\theta \cos\psi +\sin\phi \sin\psi \right)U_{1}-k_{s}\left(v_{x}-w_{x}\right)}{m}$$

(11) $$\frac{d^{2}y_{e}}{dt^{2}}=\frac{\left(\cos\phi \sin\theta \sin\psi -\sin\phi \cos\psi \right)U_{1}-k_{s}\left(v_{y}-w_{y}\right)}{m}$$

(12) $$\frac{d^{2}z_{e}}{dt^{2}}=\frac{\left(\cos\phi \cos\theta \right)U_{1}-k_{u}\left(v_{z}-w_{z}\right)}{m}-g$$

(13) $$U_{1}=b\sum_{1}^{4}\Omega_{i}^{2}$$

where $U_{1}$ is the sum of the forces of all propellers, $\Omega_{i}$ is the rotation speed of *i*-th propeller, and *b* is the thrust coefficient, $\left(w_{x},w_{y},w_{z}\right)$ are the Cartesian local wind speeds respect to the same earth-fixed frame, *g* the acceleration due to gravity, *m* is its mass, and $\left(k_{s},k_{u}\right)$ are the horizontal and vertical friction coefficients. The wind prediction is obtained from the WM and the drone’s parameters from the APM (as introduced in Section 3.4).

With these equations, each of the previously explained physical laws are integrated obtaining a trajectory prediction for the Flight Plan. A full study of the effect of the constraints is made in [69]. In our case we constraint the problem defining the flights by the following QR-AIDL instructions: horizontal speed law (implementing horizontal accelerations, horizontal decelerations and constant horizontal velocity), vertical speed law (implementing vertical accelerations, vertical decelerations and constant vertical speed), 3D speed law (implementing oblique accelerations, oblique decelerations and constant oblique speed) and yaw law (implementing yaw turns).

Finally, when the predicted trajectory is calculated it is sent to the Flight Generator module, as was described in Figure 3.

#### 3.4.3. Flight Generator: Simulated Video/Sensing Generation

Once the Trajectory has been calculated and sampled we use it to generate a simulated flight video with the predicted mission, simulating the drone camera view along the mission. This simulation will be useful to know if the distance to the objective will be short enough in order to get pictures of a specific part of the target object. In order to be able to send this video with very low bandwidth consumption to the pilot app we built a Unity-based cross-platform player. This cross-platform player is embedded both in the MDS user HMI and in the pilot App HMI. From the already explained predicted trajectory, 3D models of the mission objective, and the drone’s camera calibration (according to [73]), this player is able to simulate in real time the dynamics of the drone (by sample interpolation), the camera orientation and its point of view, and use it to locate virtual cameras in the correct relative position and attitude with respect to the scenario 3D models. Moreover, an additional video showing the relative position of the drone from the take off position (near the human pilot initial position) is also produced in order to assist pilot to understand the mission. Results showing these simulated videos can be found in Section 4.

#### 3.4.4. Flight and Measurement Plan Translations

All previous calculations are performed offline, before the flight is performed, to ease mission design and make all human actors in the inspection share a common view. In order to actually implement the flight in an autonomous way, the Flight Plan, calculated as a series of waypoints and legs, with associated measurement processes, and drone yaw and camera tilt constraints, are translated into languages understandable by the drone autopilot/FCS and measurement control systems.

In our system we have implemented two different translation subsystems. The first one has been implemented in a DJI S1000 drone with a specially tailored optical camera plus thermal camera payload controlled through a NUC system onboard, capable of broadcasting video, still pictures and thermal images in real time, and connected to GPS and IMU sensors. Another translation system has been developed for much smaller and simpler Parrot Bebop 2 drone.

In the case of our implementation for DJI drone, flight related instructions are implemented using DJI SDK to control the autopilot from our flight plan data models, while we send both the flight plan and the measurement plan to the NUC through WiFi, so it may trigger measurement once each waypoint is reached.

**Figure 6:**
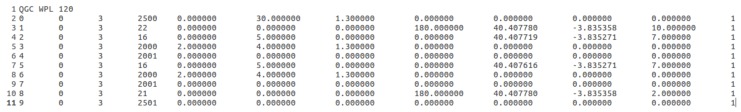
Mavlink example.

The Parrot Bebop 2 [43] drone is controllable through MAVLink [74] or Micro Air Vehicle Link, which is a protocol for communicating with small unmanned vehicle. It is based on a very lightweight, header-only message marshalling library [75], which allows to codify both flight plan related instructions and measurement triggering instructions in a time ordered sequence. For example, a Flight Plan with two waypoints would be defined by a set of consecutive MAVLINK instructions, which at very high level could be: Take off—Start video capture—Go to a waypoint (with yaw constraint)—Start/finish picture capture—Go to another waypoint (with yaw constraint)—Start/finish picture capture—End video capture—Land. An example of a MAVLink file is shown in Figure 6. The first column represents the sequence of the mission item, whereas the second and the third columns are internal parameters of MAVLink. The fourth column is the instruction code, and in the example the values that this column takes follow the pattern described in the previous list. Next columns contain the different parameters that are needed for each instruction. In the movement instructions (take off, land and go to a waypoint) 3D position of a waypoint and its yaw are provided. Meanwhile, in measurement related instructions (delay, video and picture capture) the parameters used are the time of a delay in the waypoint, frame per seconds of the video capture or the resolution in megapixels, number of pictures to take, etc. MAVLink instructions are uploaded to the drone through a FTP connection on the WiFi network created by the drone. Then, the drone is instructed to follow this MAVlink translated flight and measurement plan.

### 3.5. Drone Flight and Measurement Plan Execution

The actual autonomous execution of the flight and measurement processes is controlled by the different drones using different approaches:

- For the Bebop 2 drone, the autopilot/FCS and measurement control systems are integrated and the system just follows the aforementioned instructions in the same order they are provided, controlling the different hardware components of the drone (basically motors and camera), and georeferencing the trajectory by making use of the on-board GPS system.
- For the DJI drone the procedure is more complex. The flight plan is executed using DJI drone SDK, while the measurements are taken by the cameras while triggered by the NUC. The NUC triggers picture taking, according to measurement plan, once it decides an inspection waypoint in the flight plan is reached, and the aircraft yaw is coherent with yaw constraints at that waypoint. To reduce GPS and IMU measurement errors a Kalman filter is used, along with some integrity tests to discard inertial and GPS measurement outliers. Gimbal tilt control in this drone is not possible (by automatic means), which imposes some limits on the potential illumination strategies.

## 4. Results

Representative uses and results of the overall system will be included next. Specifically, in Section 4.1 we will show some examples of complex representative mission definition processes. Later, Section 4.2 will show trajectory calculation results, including simulated flight video capture results, and compare them with actual flights, both for manual and automated operation.

### 4.1. Mission Creation Process Results

The first step is to define the desired target central location, using the map tool described in Section 3.3. In order to do this, it is possible to update the map scope (by usual gestures such as drag and pan). Then, as shown in the panel A of Figure 2 other mission details can be introduced, such as a text description and initial and final waypoints (for take off and landing), which also can be defined by clicking on the map. Initial and final waypoints can be updated.

The second step is to select a collection of basic inspections from the list described in Section 3.3. The general process is the same for all the inspections (with some modifications), but to illustrate the example, a polygon inspection is used as example. When the user selects an inspection, the map allows the user to set its position in the 3D map. Once the inspection is located at the desired position the interface offers the possibility to update it graphically through geometric transformations based on placeholders dragging, and through text input. Then, the user must fill in the data in a panel to complete the definition of the measurement processes for the inspection, as defined in Section 3.3.

**Figure 7:**
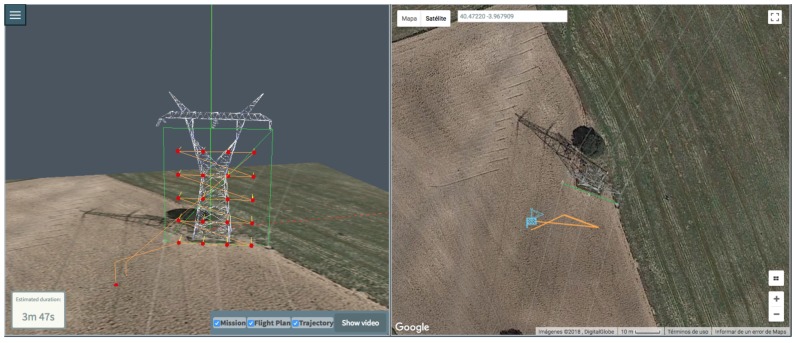
Generated Flight Plan and Trajectory views: (**left**) lateral (**right**) zenit.

After creating all the basic inspections in the mission, the next step is to actually generate the Flight Plan and the Trajectory. When the user clicks on the “Generate mission” button in Figure 2, the tool generates both of them and updates the 3D view and the map properly, as shown in Figure 7. On its left side the created inspection shape can be seen in the 3D view with green lines. The red dots represent the calculated waypoints. Each waypoint has an associated yellow line, which indicate the bearing direction of the sensors. The orange line represents the calculated Trajectory to be followed by the drone to accomplish the mission. On the other hand, the right part of the interface holds the interactive map that shows the 2D representation of the mission and trajectory.

**Figure 8:**
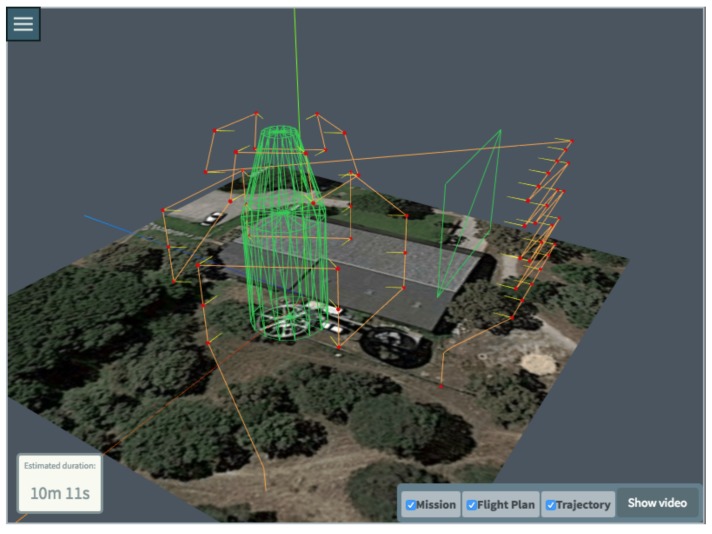
A more complex mission with multiple inspections.

Figure 8 shows a more complex mission example to illustrate the potential of the tool, that allows the user to generate a mission with an arbitrary number of basic inspections. For this example, multiple basic inspections were defined: a polygon, a vertical cylinder and a truncated cone, emulating the shape of a more complex interest shape. The tool offers the possibility to iteratively edit a mission, so more basic inspection can be added or removed, and measurement processes may also be changed.

Flight duration can be seen at the bottom-left corner of the representation. This helps the MDS user to have a perception of the real flight duration that the flight will take, and to take into account the battery duration constraints and decide if the flight should be split.

### 4.2. Mission Calculation and Comparison with Real Flights Execution

To test our solution in the field, we performed two real inspections with our two drones.

**Figure 9:**
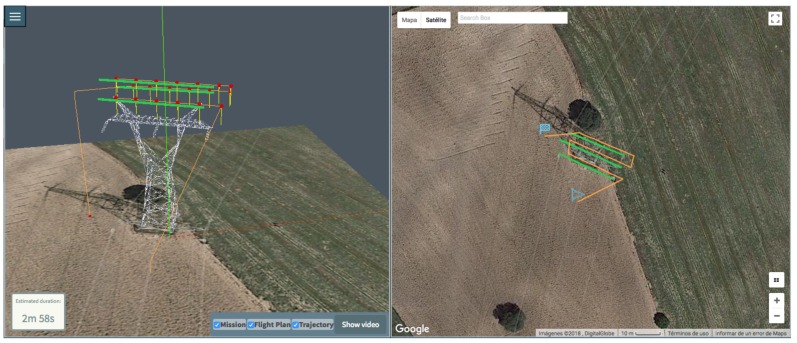
Generated Flight Plan and Trajectory for high voltage tower: (**left**) lateral, (**right**) zenit.

We used the DJI drone to inspect part of a high voltage electric distribution tower. The inspection consists of three horizontal lines with different heights over the tower: the central one is higher to avoid the upper part of the tower. The objective is to take optical and thermal pictures of tower isolation elements, cables and structure, to look for potential defects or anomalies. Figure 9 shows two 3D perspectives for the calculated trajectories and flight plans.

**Figure 10:**
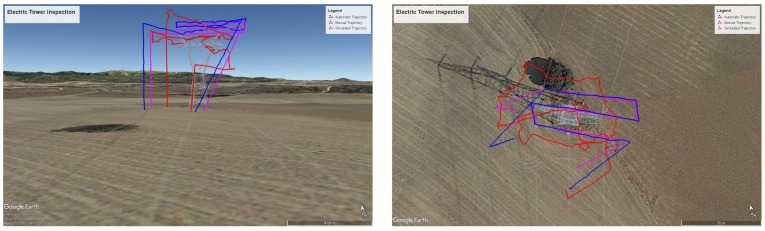
Manual (red), automated (blue) and MDS simulated/predicted (magenta) 3D trajectory: (**left**) lateral, (**right**) zenit.

We performed the inspection twice. The first time we did it manually, with the drone being piloted by a certified drone pilot, who received a description of the overall mission to be performed and also the 3D view of the mission, through the pilot App. Afterwards, the flight was also performed using the automation process described in the paper. In Figure 10 the manual, automated and MDS simulated/predicted trajectories are depicted. With respect to the manual trajectory, it is evident it has much bigger errors. There is some initial unexpected pattern as the drone was kept hovering for a small time interval as the payload was remotely accessed to manually control the camera tilt. During the automated flight this was also done manually, but during climbing.

**Figure 11:**
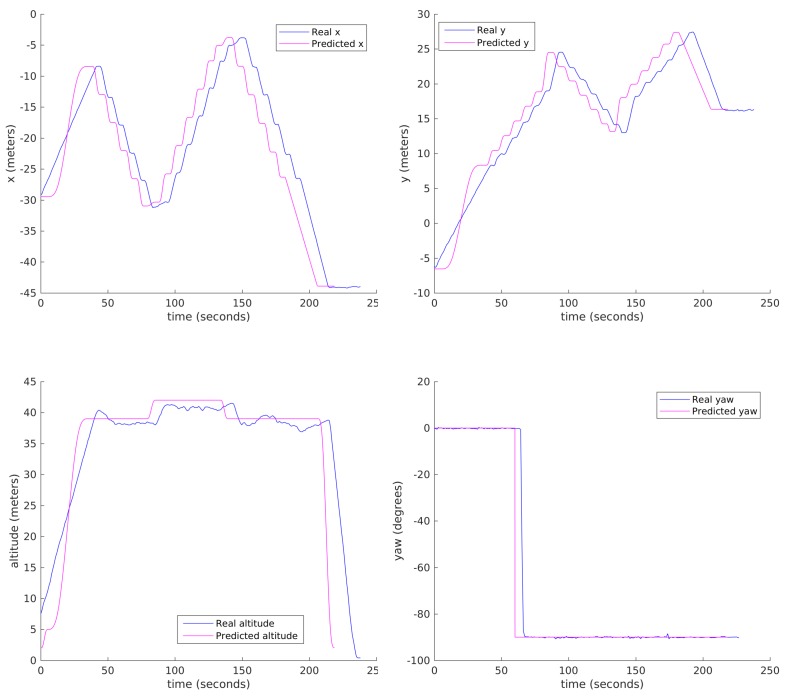
Automated execution vs MDS Simulated/Predicted results (x (**top-left**), y (**top-right**), altitude (**bottom-left**) and yaw (**bottom-right**)).

Figure 11 compares the variation along time of the real automated flight (blue) and MDS simulated/predicted (magenta) trajectories. The proposed trajectory prediction is in general correct, although there is some need to improve it along climbing sections, specially just after takeoff.

**Figure 12:**
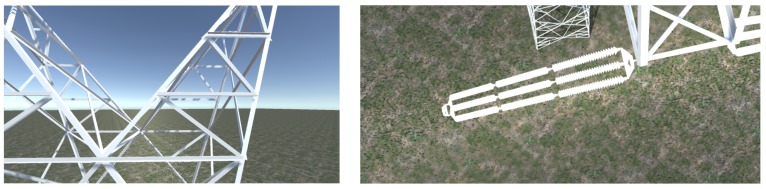
Simulated flight video results ((**left**): lateral, (**right**): zenit).

For this scenario, Figure 12 shows the simulated synthetic view obtained from the drone’s camera at a certain instant while the aircraft is moving towards the top of the tower, and also the zenith view from the drone overflying a certain area of the tower isolators.

**Figure 13:**
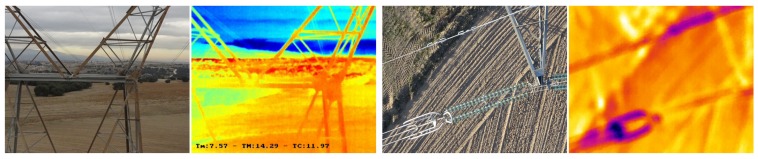
High voltage tower pictures, from left to right: optical-lateral, thermal-lateral, optical-zenit and thermal-zenit.

Figure 13 contain pictures taken from the drone both through the optical camera and from the thermal camera, while the aircraft is moving towards the top of the tower, and overflying a certain area of the tower isolators, respectively. It may be seen the images are similar to the ones predicted by the MDS (the 3D models are not exact).

**Figure 14:**
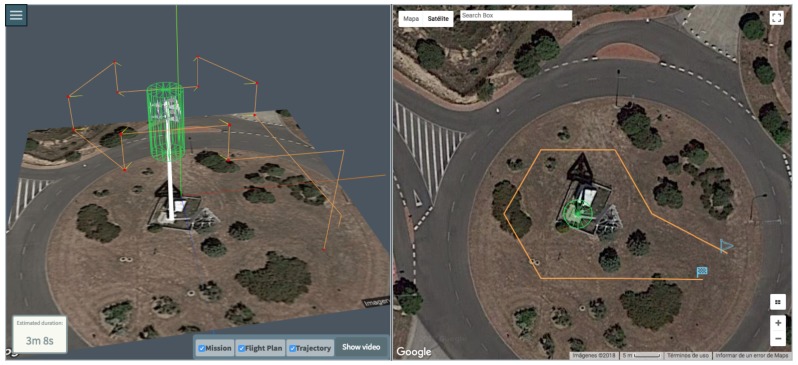
Mission of the inspection of a telecommunication antenna.

Meanwhile, with the Bebop 2 drone, we performed the inspection of a cell tower, whose mission, generated flight plan and predicted trajectory can be seen in Figure 14.

**Figure 15:**
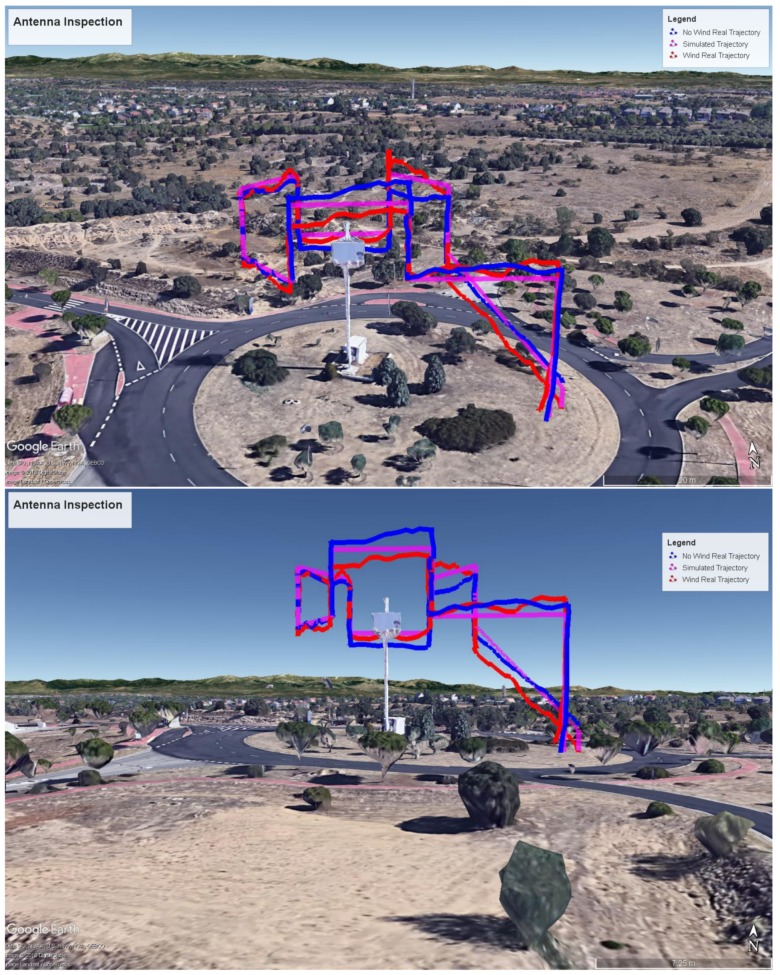
Two different perspectives of real (little wind, windy conditions) and MDS predicted/simulated 3D trajectory.

To check the validity of our results, two automated flights in two different days were performed: one with little wind (smaller than 5 m/s), and another one with stronger wind (in the order of 10–15 m/s, with gusty conditions). In both cases, for the windy and for the not windy day, zero wind was assumed. Figure 15 shows the trajectories followed by the drone without wind (blue), the trajectory at a windy day (red) and the MDS trajectory prediction/simulation (magenta).

**Figure 16:**
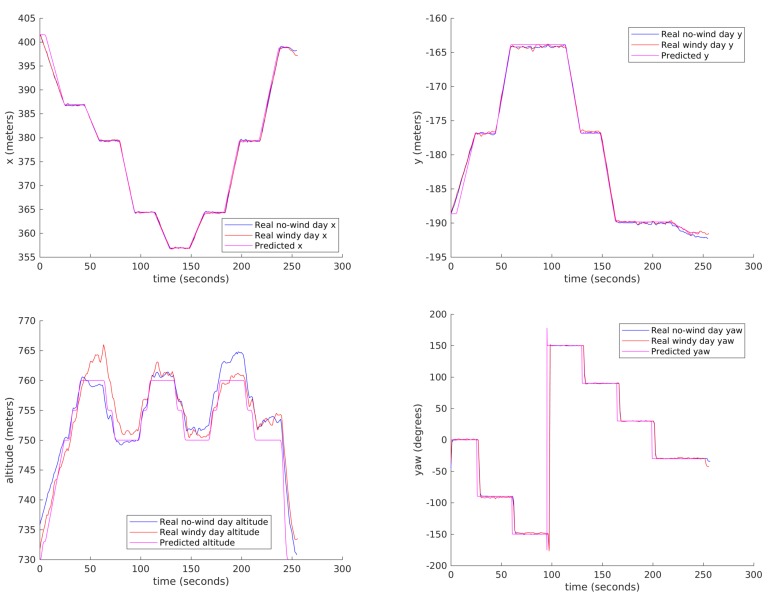
Mission execution and simulated results (x (**top-left**), y (**top-right**), altitude (**bottom-left**) and yaw (**bottom-right**)).

Figure 16 shows some results comparing the real dynamic parameters and the predicted ones, specifically the (x, y, z) Cartesian position and the yaw angle respectively, both real (measured) telemetry data for the day with little wind (blue), the same data for the day with stronger wind (red) and MDS predicted data (magenta). It can be observed that our trajectory predictor is quite accurate, providing very similar results to the real flight except for two differences: there is a time offset due to the slower take off of the real drone, and there are also differences in the altitude because of the lower quality of the drone altitude control. The first of the problems might be alleviated by improving take-off modelling in QR-AIDL instructions. The trajectory is quite similar with and without wind.

**Figure 17:**
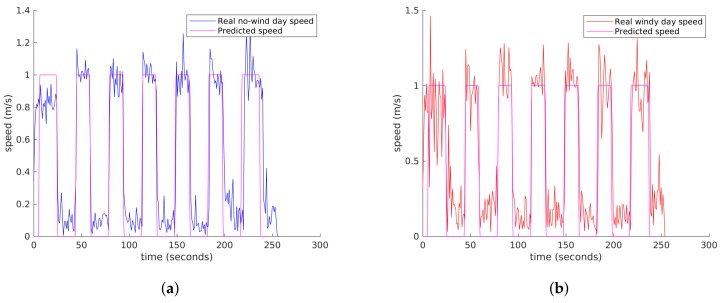
Comparison of groundspeed between normal conditions and a day of strong wind. (**a**) Low wind real and MDS predicted groundspeed; (**b**) Strong wind real and MDS predicted groundspeed.

The biggest difference between these two real flights is the groundspeed stability, as it can be observed in Figure 17. In the day with strong wind, the speed undergoes greater changes than in a normal day, as stabilisation is more problematic.

**Figure 18:**
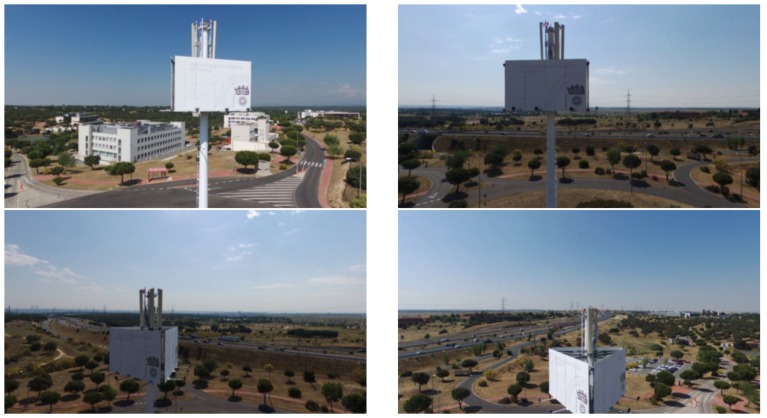
Four in-flight taken pictures from different perspectives along the flight.

The photographs taken by the drone at the mission waypoints during actual flight execution can be seen in Figure 18.

## 5. Discussion and Conclusions

This paper describes the architecture of a multipurpose drone-oriented Mission Definition System. The system has been designed to facilitate the definition of flight missions for different type of automated operations through visual tools, in order to overcome the limitations of the basic tools provided by drones’ manufacturers and to facilitate users and pilots to share a common view of the operation. The MDS provides the interfaces to implement automated operations for different UAV autopilots and sensing payloads. The tool defines a set of predefined basic inspection modes that help easily define parts of a wide number of mission operations, as missions are configurable by concatenation of those basic geometries. Prior to the actual flight, the System enables to play a 3D simulated flight, to help guarantee the user that the information retrieved during the operation will be the needed one. Once the mission is defined, the MDS is ready to provide flight scripts for the autopilot enabling the flight to be implemented in automated mode. The operation of the MDS is exemplified through different real inspection flights, which typify the differences between the actual flights operating the drone manually against using the automated tool, and demonstrate the viability of the tool to support this kind of operations. To our experience with operators in inspections, this approach of flight specification and implementation provides satisfactory outcomes both for users and pilots, and is a step forward to the automatic operation of drone fleets.

Our results show that in general the trajectory predictions are good enough for the intended purposes, even for windy situations, but there is some room for improvement of flight guidance modelling through QR-AIDL, specially for climbs just after takeoff.

Another feature of our platform, not discussed in the paper and currently being implemented is the capability to enable real-time remote visualization of drone’s telemetry and sensors (including video streaming), in order to make possible that the user monitors the flight execution and results in a set of HMIs integrated with those of the Mission Definition System. Additionally, the presented system is currently being evolved to be part of a drone fleet management tool. An important improvement would be connecting this management tool to airspace management and flight authorisation authorities, by converting the missions to formats compatible to UAV Traffic Management (UTM) systems.
